# Clinical Characteristics of Young Type 2 Diabetes Patients with Atherosclerosis

**DOI:** 10.1371/journal.pone.0159055

**Published:** 2016-07-08

**Authors:** Wenjia Yang, Xiaoling Cai, Xueyao Han, Linong Ji

**Affiliations:** Endocrinology and Metabolism Department, Peking University People’s Hospital, Beijing, China; Beijing Key Laboratory of Diabetes Prevention and Research, CHINA

## Abstract

**Objective:**

The prevalence of type 2 diabetes is increasing rapidly in the young population. The clinical characteristics and risk factors for young type 2 diabetes patients with atherosclerosis are not fully explicated. The aim of the present study was to investigate various clinical and biochemical characteristics of young type 2 diabetic patients with atherosclerosis.

**Design and Methods:**

This was a cross-sectional study. The study involved 2199 hospitalized patients with type 2 diabetes. The young patients were classified into the atherosclerotic group or the non-atherosclerotic group, and we also enrolled an older group with peripheral atherosclerosis disease and an age of at least 45 years. Comparisons were made between the different groups to investigate the cardiovascular and metabolic risk profiles of young type 2 diabetes patients with atherosclerosis. We also used logistic regression models to assess the atherosclerosis risk factors for young patients.

**Results:**

Compared to older type 2 diabetes patients with atherosclerosis, young patients with atherosclerosis had more deleterious profiles of weight and hyperlipidemia. Only age and diabetes duration were found to be significant independent risk factors for atherosclerosis in young patients. The ratio of the presence of atherosclerosis in the lower extremity arteries alone was significantly higher in young patients than older patients (26.4% vs. 14.0%, P = 0.000).

**Conclusion:**

Young type 2 diabetes patients with atherosclerosis have more adverse cardiovascular risk profiles and inadequate control of these risk factors. Lower extremity examination is of high importance in young patients.

## Introduction

The prevalence of type 2 diabetes in young patients has increased significantly in recent years with rapid growth of the diabetic population [1,2]. The results of a nationwide epidemiological study in China demonstrated that in 2007, the prevalence of diabetes was 3.2% among persons who were 20 to 39 years old [3].An epidemiological study performed in the UK showed that the incidence of type 2 diabetes in those aged younger than 30 years rose from 0.1 to 4.9 per 100,000 between 1991 and 2006 [1]. It is well known that macrovascular complications correlated with atherosclerosis are the major threat for type 2 diabetic patients. A population-based study in Sweden showed a threefold excess mortality rate in young type 2 diabetic patients (aged 15–34 years), and the most common underlying cause of death in these patients was circulatory disease [4].With increases in age and prolongation of disease duration, the time exposed to risk factors is longer for young diabetes patients, which puts them at higher risk of developing atherosclerosis. In this case, diabetes in young patients has become a major public health problem, and strategies aimed at preventing complications are needed.

Benhalima’s cross-sectional study showed that in type 2 diabetic patients younger than 40 years old, 71.8% had cholesterol>4mmol/L, 54.9% had triglycerides>1.7mmol/L, and 70% had HbA1c levels>7.0%, suggesting they had inadequately treated risk factors [5].While Gunathilake’s study, which focused on young type 2 diabetes patients, suggested that the proportions of adverse cardiovascular disease risk profiles for young patients were similar to the older patients. However, this study excluded patients with history of cardiovascular disease and patients received medications for lipid or blood pressure, potentially bringing bias to the study [6]. Therefore, we tested the hypothesis that compared with older patients, young type 2 diabetes patients with atherosclerosis might have more cardiovascular risk factors. The aim of this retrospective study was to find the clinical and biochemical characteristics of young type 2 diabetes patients with atherosclerosis in order to prevent and delay the development of atherosclerosis in young patients.

## Methods

### Patients

This study began in Feb. 2015. By searching the inpatient database at Peking University People’s Hospital, we identified 2199 type 2 diabetic patients who were hospitalized for treatment in the ward of the Department of Endocrinology and Metabolism of Peking University People’s Hospital from Jan. 2009 to Dec. 2014. All included patients were aged 20 to 85 years old.

### Ethics Statement

The data were analyzed anonymously in this retrospective study, therefore, there was no need for informed consent. The ethics committee of Peking University People’s Hospital has approved this retrospective study. All patients were admitted to the Department of Endocrine and Metabolism of our hospital, and during the day of their admission, they signed the consent form allowing their information and past data to be stored in the hospital database and used for research, and this consent form was also approved by the ethics committee of Peking University People’s Hospital. All patients’ information was coded by database staff who were not involved in data extraction and analysis. No authors in this study had access to patient identifying information during or after data extraction from this database. No authors in this study were involved in de-identifying the patient data.

### Variable assessment

The following measurements were collected from the electronic database for each patient: age, sex, diabetes duration, body mass index (BMI), systolic blood pressure (SBP), diastolic blood pressure (DBP), waist circumference, and fasting glucose, postprandial 2-hour glucose, glycosylated hemoglobin (HbA1c), fasting plasma glucose (FPG), postprandial plasma glucose (PPG), total cholesterol (TC), low-density lipoprotein (LDL-C), high-density lipoprotein (HDL-C), triglyceride (TG), and uric acid (UA) levels. The patients received retinal photography by the same trained photographer taking two 45° digital retinal images per eye. The retinal images then were transferred to the ophthalmologist for grading. Urine samples were taken in three consecutive days on first morning void. Average urinary albumin-to-creatinine ratio (ACR) was calculated. Diabetic nephropathy was defined as having persistent urinary albumin to creatinine ratio ≥ 30mg/g after excluding other causes of kidney damage, urinary system infection and blood in urine.

### Peripheral atherosclerosis

Ultrasound of the carotid artery and lower extremity artery were examined for each patient. Carotid atherosclerosis was defined as the presence of atherosclerotic plaques, stenosis and occlusion in any aterial segments of the common carotid arteries, the bifurcation, the external carotid arteries and the internal carotid arteries. The presence of plaque, stenosis and occlusion in any segments of common femoral artery, profunda femoris artery, superficial femoral artery, popliteal artery, anterior tibial artery, posterior tibial artery and peroneal artery were defined as lower limb atherosclerosis.

### Patients grouping

The included patients were divided into the young group and the older group by being under or over the age of 45 years. Young diabetic patients were defined by being no more than 45 years old, and older diabetic patients were defined as being more than 45 years old. Patients were further divided into the atherosclerosis group and non-atherosclerosis group by the diagnosis of peripheral atherosclerosis using ultrasound.

### Statistical analysis

SPSS version 19.0 was used for statistical analyses. Continuous data were expressed as the mean±standard deviation if normally distributed, and non-parametric variables were expressed as median and values of 25^th^ and 75^th^ percentiles. Categorical data were expressed as percentage. To compare variables between the two groups, an independent samples t-test was used for normally distributed continuous variables. Comparisons between the four groups were performed using analysis of variance (ANOVA). Mann-Whitney U tests were used for non-parametric variables and Chi-squared tests were used for categorical variables. All P values were two-sided, and P values less than 0.05 were considered to indicate statistical significance. Binary logistic regression analysis was used to assess the odds ratio of each risk factor for atherosclerosis in young patients.

## Results

### Characteristics of hospitalized young type 2 diabetes patients with atherosclerosis

Among the 2,199 included patients, 391 (17.8%) were young patients and 1,808 (72.2%) were older patients; 1,802 (81.9%) patients were diagnosed with peripheral atherosclerosis, and 397 (18.1%) subjects were non-atherosclerosis patients; 48.1% young patients and 87.9% older patients were diagnosed with peripheral atherosclerosis respectively. The average age for young type 2 diabetes patients with atherosclerosis was 42.0 (27, 45) years, the duration of diabetes was 4.0(0.1, 19.0) years, 82.3% were male, the average BMI was 26.7±4.5 kg/m^2^, and the average HbA1c level was 9.3±2.2%. (Table 1).

**Table 1:** Clinical characteristics and laboratory measurements for the young atherosclerosis group, the young non-atherosclerosis group, older atherosclerosis group and older non-atherosclerosis group.

	All (n = 2199)	Young atherosclerosis group (n = 186)	Young non-atherosclerosis group (n = 205)	Older atherosclerosis group (n = 1616)	Older non-atherosclerosis group (n = 192)	P value
**Age (year)**	56.0(20, 85)	42.0(27, 45)*#	36.0(20, 45)*	60.0(46, 85)#	53.0 (46, 84)	<0.001
**Male (%)**	59.8(1316)	82.3(153)#	75.1(154)	56.4(912)#	50.5 (97)	<0.001
**Diabetes duration (year)**	9.0(0.1, 47.0)	4.0(0.1, 19.0)*#	2.0(0.1, 15.0)*	10.0(0.1, 47.0)#	6.0 (0.1, 30.8)	<0.001
**BMI (kg/m**^**2**^**)**	25.6±3.7	26.7±4.5#	26.5±4.0	25.4±3.5#	25.0±3.5	<0.001
**Waist (cm, male)**	93.8±10.5	95.9±11.4#	95.8±11.6	93.2±10.1#	92.4±10.6	0.001
**Waist (cm, female)**	91.7±11.0	89.6±13.9*#	88.8±9.4*	92.4±10.8#	87.9±11.2	<0.001
**SBP (mmHg)**	131.5±17.6	128.1±14.5#	124.9±16.1	133.1±17.9#	128.4±16.8	<0.001
**DBP (mmHg)**	79.4±11.2	82.8±10.2	80.4±12.6	78.8±10.9	80.0±12.3	<0.001
**HbA1c (%)**	9.2±2.1	9.3±2.2*	10.0±2.2*	9.0±2.1	9.2±2.1	<0.001
**FPG (mmol/L)**	8.0±2.9	8.2±3.0	8.5±3.2	7.9±2.8	8.1±2.9	0.051
**PPG (mmol/L)**	12.4±4.2	12.9±4.4	12.3±4.2	12.4±4.1	12.3±4.1	0.478
**TC (mmol/L)**	4.67±1.18	4.87±1.47	4.75±1.15	4.65±1.15	4.62±1.08	0.065
**TG (mmol/L)**	1.52 (1.07, 2.20)	1.86 (1.32, 2.68)#	1.77 (1.16, 2.76)	1.47 (1.05, 2.13)#	1.41 (0.94, 2.05)	<0.001
**LDL-C (mmol/L)**	2.71±0.88	2.87±0.91	2.73±0.89	2.70±0.87	2.67±0.87	0.078
**HDL-C (male, mmol/L)**	0.98±0.30	0.92±0.20#	0.90±0.24	1.00±0.32#	1.00±0.32	<0.001
**HDL-C (female, mmol/L)**	1.13±0.36	1.12±0.48	1.03±0.28	1.13±0.37	1.14±0.29	0.304
**UA (male, μmol/L)**	340.8±89.1	361.6±88.4	359.4±97.6	335.4±87.0	328.1±87.4	0.120
**UA (female, μmol/L)**	295.7±88.4	305.3±95.3	284.9±89.2	298.1±87.7	279.5±89.8	0.207
**ACR (mg/g)**	8.1 (3.7, 23.0)	6.3 (3.8, 16.0)#	6.1 (3.8, 13.7)	8.9 (3.8, 28.0)#	6.5 (3.2, 16.2)	0.026

*significant difference between the young atherosclerosis group and the young non-atherosclerosis group (P<0.01).

^#^ significant difference between the young atherosclerosis group and the older atherosclerosis group (P<0.01).

BMI, body mass index; SBP, systolic blood pressure; DBP, diastolic blood pressure; HbA1c, glucosylated hemoglobin; FPG, fasting plasma glucose; PPG, postprandial plasma glucose; TC, total cholesterol; TG, triglyceride; LDL-C, low-density lipoprotein; HDL-C, high-density lipoprotein; UA, urine acid; ACR, urine albumin-to-creatinine ratio.

### Difference in demographic data and metabolic profiles between the young atherosclerosis group, young non-atherosclerosis group and older atherosclerosis group

Compared to young non-atherosclerotic patients, young atherosclerotic patients were more likely to have older age, longer diabetes duration, larger waist size in females and lower HbA1c levels, while the total cholesterol, LDL-C, HDL-C and TG levels were similar between these two groups.

Compared to the older atherosclerotic patients, young patients with atherosclerosis were more likely to be male, have significantly shorter diabetes durations, and have higher BMI. Higher TG and lower HDL-C in males were also observed in young atherosclerotic patients (Table 1).

### Difference in microvascular complications between the young atherosclerosis group, young non-atherosclerosis group and older atherosclerosis group

Young patients with atherosclerosis were at significantly higher risk of developing diabetic retinopathy compared with young non-atherosclerotic diabetic patients, especially with non-proliferative diabetic retinopathy (P<0.01). There was no difference in diabetic nephropathy between these two groups.

While older atherosclerotic patients were at significantly higher risk of developing both diabetic retinopathy and diabetic nephropathy than young patients with atherosclerosis, the proportion of patients with non-proliferative diabetic retinopathy, macroalbuminuria and chronic kidney disease was particularly higher in the older atherosclerosis group (P<0.01; Table 2).

**Table 2:** Difference in microvascular complications between the young atherosclerosis group, the young non-atherosclerosis group and the older atherosclerosis group.

	All (n = 2199)	Young atherosclerosis group (n = 186)	Young non-atherosclerosis group (n = 205)	Older atherosclerosis group (n = 1616)	Older non-atherosclerosis group (n = 192)	P value
**Diabetic Retinopathy (%)**	22.6 (496)	15.1 (28)*#	6.3 (13)*	26.9 (434)#	10.9 (21)	<0.001
**Non-proliferative Diabetic Retinopathy (%)**	15.1 (322)	9.9 (18)*#	3.0 (6)*	18.2 (285)#	6.8 (13)	0.018
**Proliferative Diabetic Retinopathy (%)**	5.2 (111)	3.8 (7)	1.5 (3)	5.9 (93)	4.2 (8)	0.040
**Diabetic Nephropathy (%)**	21.9 (460)	15.9 (28)#	17.0 (33)	24.2 (375)#	13.0 (24)	<0.001
**Microalbuminuria (%)**	14.6 (320)	13.6 (24)	13.4 (26)	16.2 (251)	10.3 (19)	0.146
**Macroalbuminuria (%)**	6.4 (140)	2.3 (4)#	3.6 (7)	8.0 (124)#	2.7 (5)	0.001
**Chronic Kidney Disease (%)**	5.4 (119)	1.1 (2)#	0.0 (0)	7.1 (111)#	3.2 (6)	<0.001
**End-stage Renal Disease (%)**	—	—	—	—	—	—

*significant difference between the young atherosclerosis group and the young non-atherosclerosis group (P<0.01).

^#^ significant difference between the young atherosclerosis group and the older atherosclerosis group (P<0.01).

### Risk factors for young type 2 diabetic patients with atherosclerosis

In binary logistic regression, young patients with relatively older ages and longer diabetes durations were at greater odds of developing atherosclerosis (P<0.01). Sex, BMI, waist circumference, SBP, HbA1c, TG, LDL-C, ACR, and diabetic retinopathy were not independent atherosclerosis risk factors (Table 3).

**Table 3:** The odds ratio of clinical characteristics and laboratory measurements for young atherosclerosis patients versus young non-atherosclerosis patients.

	OR	95%CI		p
**Age (year)**	1.139	1.089	1.192	<0.001
**Male (%)**	0.486	0.242	0.978	0.043
**Diabetes duration (year)**	1.101	1.030	1.178	0.005
**BMI (kg/m**^**2**^**)**	1.086	0.959	1.228	0.193
**Waist (female) (cm)**	1.021	0.967	1.079	0.453
**Waist (male) (cm)**	0.996	0.971	1.022	0.763
**SBP (mmHg)**	1.006	0.989	1.023	0.483
**HbA1c (%)**	0.977	0.858	1.113	0.726
**TG (mmol/L)**	1.015	0.908	1.135	0.791
**LDL-C (mmol/L)**	1.325	0.977	1.797	0.070
**ACR (mg/g)**	1.000	0.999	1.001	0.458
**DR (%)**	2.164	0.825	5.680	0.117

The odds ratio (OR) and its 95% confidence interval of each parameter versus young non-atherosclerosis patients were analyzed by binary logistic regression. BMI,body mass index;SBP, systolic blood pressure;HbA1c,glucosylated hemoglobin;TG,triglyceride;LDL-C,low-density lipoprotein;ACR, urine albumin-to-creatinine ratio; DR,diabetic retinopathy.

### Atherosclerosis distribution in the young atherosclerosis group, young non-atherosclerosis group and older atherosclerosis group

The assessment of peripheral atherosclerosis distribution using vascular ultrasound in different groups showed that the incidence of atherosclerosis in the carotid artery and lower extremity were 30.3% and 46.6% in young patients and 78.5% and 82.2% in older patients, respectively. Moreover, 17.8% of the young patients and 11.1% of the older patients developed atherosclerosis only in the carotid artery, which showed no significant difference between the young group and older group. However, the incidence of atherosclerosis only found in a lower extremity artery was significantly higher in young patients (46.5% and 15.0%, respectively). However, the incidence of atherosclerosis found both in the carotid and lower extremity artery was significantly higher in older patients (35.6% and 72.9%, respectively).

## Discussion

At present, most studies focused on the risk factors of atherosclerosis in general type 2 diabetes populations, but investigations about the features in young patients were scarce. Therefore, we carried out this retrospective study to access the characteristics of young type 2 diabetic patients with atherosclerosis comprehensively, and tried to explore the risk factors in the development of atherosclerosis in Chinese hospitalized type 2 diabetics. The present study showed that young atherosclerotic type 2 diabetic patients tended to be older and have longer diabetes durations than young non-atherosclerotic patients. Compared to older atherosclerotic patients, they have a risk of higher BMI, waist circumstance and poorer control of blood lipid parameters. Glucose control was comparable between young and older atherosclerotic patients. The risk of diabetic retinopathy was higher for young atherosclerotic patients than for young non-atherosclerotic patients, but it was lower than that for older atherosclerotic subjects. Age and diabetes duration were independent risk factors for atherosclerosis for young type 2 diabetic patients.

In this study, young atherosclerotic and young non-atherosclerotic patients showed no significant differences in the levels of plasma glucose and lipid profiles except for the waist in female patients. Only age and diabetes duration were independent risk factors in logistic regression analysis, which were widely known risk factors in the development atherosclerosis. Nevertheless, young patients will have longer exposure to these risk factors due to young age at diagnosis compared to those diagnosed at older age. Furthermore, some studies have demonstrated that the increments in cardiovascular disease, diabetic retinopathy and diabetic nephropathy incidences were higher in young type 2 diabetes patients than older subjects even with the same duration [7].Therefore, we need to fully recognize and control these risk factors. In addition, whether genetic susceptibility plays any role in the development of atherosclerosis in young patients still needed further studies.

Our study demonstrated that the presence of atherosclerosis in lower extremities was more common than that in the carotid artery in young subjects. However, the presence of atherosclerosis in the carotid artery and lower extremities was similar in older patients. Carotid artery ultrasound is one of the most common methods used for atherosclerosis disease screening. Atherosclerosis in the carotid artery is considered to be the “window” of atherosclerosis disease in the whole body. Craven’s case controlled study showed a significant relation between carotid artery atherosclerosis and coronary heart disease [8]. Bots’s study suggested an association between intima-media thickness and lower extremity atherosclerosis [9]. Another study carried out with older patients with hypertension indicated that carotid artery atherosclerosis is significantly related to lower extremity atherosclerosis. However, the correlations between manifestation of atherosclerosis in the carotid artery and other regions are not that definite [10,11], and few studies have been aimed at type 2 diabetes. Li et al. suggested that the incidence of atherosclerosis in the carotid artery was significantly lower than that in a lower extremity for newly diagnosed type 2 diabetes patients (mean age 52 years; 29.9% vs. 55.2%, respectively). Yu’s study also showed similar results [12]. Combined with the results in this study, we inferred that the development of atherosclerosis in lower extremities might occur earlier than that in the carotid artery. The presence of atherosclerosis in lower extremities was found to be associated with cardiovascular disease. Sosnowski’s study showed that atherosclerotic lesions detected by ultrasound in the common femoral arteries strongly indicated coexistence of severe coronary artery disease [13]. Therefore, we need to recognize the importance of lower extremity screening for type 2 diabetes patients in clinical practice, especially for young patients.

There were some limitations in our study. First, this study was designed as a single-center, cross-sectional study. The included subjects were hospitalized patients with complicated conditions and complications; thus, there might be selection bias in the study. Moreover, the sample size of our study might not fully reflect the characteristics of young patients.

## Conclusion

In summary, young type 2 diabetes patients with atherosclerosis have more adverse cardiovascular risk profiles and inadequate control of these risk factors. Lower extremity examination is of high importance in young patients.

## Supporting Information

S1 File STROBE Checklist. (DOC)
